# Hoffa’s fat pad abnormalities, knee pain and magnetic resonance imaging in daily practice

**DOI:** 10.1007/s13244-016-0483-8

**Published:** 2016-03-21

**Authors:** F. Draghi, G. Ferrozzi, L. Urciuoli, C. Bortolotto, S. Bianchi

**Affiliations:** Radiology Institute, University of Pavia, Via Oberdan 21, 27100 Pavia PV, Italy; Department of Radiology, Guglielmo da Saliceto Hospital, Piacenza, Italy; Institute of Radiology, Second university of Naples, Naples, Italy; CIM SA, Cabinet Imagerie Médicale, Genève, Suisse

**Keywords:** Fat pad, Knee, Regional anatomy, Pathology, Magnetic resonance imaging

## Abstract

Hoffa’s (infrapatellar) fat pad (HFP) is one of the knee fat pads interposed between the joint capsule and the synovium. Located posterior to patellar tendon and anterior to the capsule, the HFP is richly innervated and, therefore, one of the sources of anterior knee pain. Repetitive local microtraumas, impingement, and surgery causing local bleeding and inflammation are the most frequent causes of HFP pain and can lead to a variety of arthrofibrotic lesions. In addition, the HFP may be secondarily involved to menisci and ligaments disorders, injuries of the patellar tendon and synovial disorders. Patients with oedema or abnormalities of the HFP on magnetic resonance imaging (MRI) are often symptomatic; however, these changes can also be seen in asymptomatic patients. Radiologists should be cautious in emphasising abnormalities of HFP since they do not always cause pain and/or difficulty in walking and, therefore, do not require therapy.

*Teaching Points*

• *Hoffa’s fat pad (HFP) is richly innervated and, therefore, a source of anterior knee pain.*

• *HFP disorders are related to traumas, involvement from adjacent disorders and masses.*

• *Patients with abnormalities of the HFP on MRI are often but not always symptomatic.*

• *Radiologists should be cautious in emphasising abnormalities of HFP.*

## Introduction

### Normal anatomy and magnetic resonance imaging appearance

There are several fat pads within the knee joint, each one interposed between the joint capsule and the synovium, and therefore intracapsular and extrasynovial [1]. Hoffa’s fat pad (HFP) is one of the three anterior fat pads, along with the anterior suprapatellar and the posterior suprapatellar (prefemoral) fat pad [2].

**Fig. 1:**
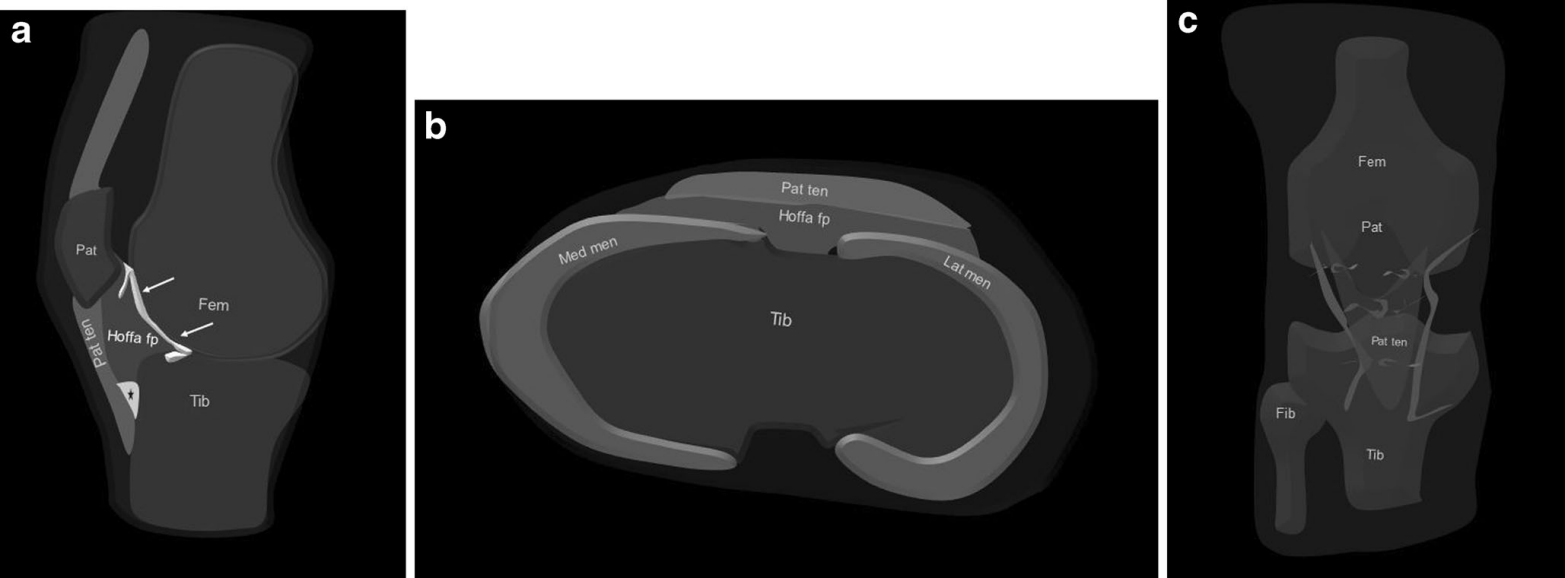
Anatomy of the HFP. The HFP (*Hoffa fp*) is limited anteriorly by the patellar tendon (*Pat ten*) and the joint capsule, superiorly by the inferior pole of the patella (*Pat*) (**a**), inferiorly by the proximal tibia (*Tib*) and the deep infrapatellar bursa (*asterisk*), and posteriorly by the synovium (*arrows*) and femur (*Fem*). It is attached directly to the anterior horns of the menisci (*Med men*, *Lat men*) (**b**). Normal vascular supply consists of two vertical arteries, posterior and parallel to the lateral edges of the patellar tendon (**c**)

**Fig. 2:**
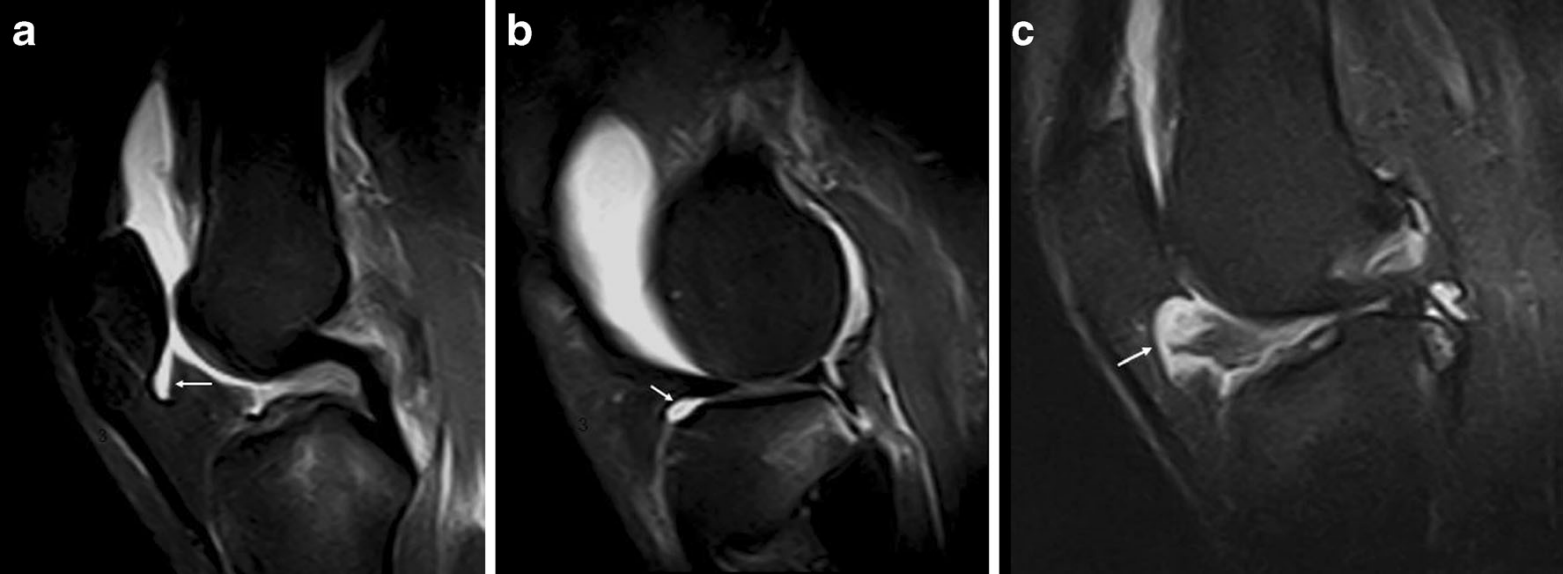
Suprahoffatic recess and infrahoffatic recess. Sagittal proton density with fat saturation MRI images show fluid in suprapatellar pouch, suprahoffatic recess (*arrow*, **a**) and infrahoffatic recess (*arrow*, **b**); a communication between the vertical and horizontal recesses may be present (*arrow*, **c**)

HFP is limited anteriorly by the patellar tendon and the joint capsule, superiorly by the inferior pole of the patella, inferiorly by the proximal tibia and the deep infrapatellar bursa, and posteriorly by the joint synovium [1, 3] (Figs. 1 and 2). A superior, vertically orientated suprahoffatic (Fig. 2a) and an inferior, horizontal infra-hoffatic (Fig. 2b) recess may be observed within the HFP [4]. A communication between the two recesses may be present (Fig. 2c). HFP is attached to the anterior horns of the menisci (Fig. 1b).

**Fig. 3:**
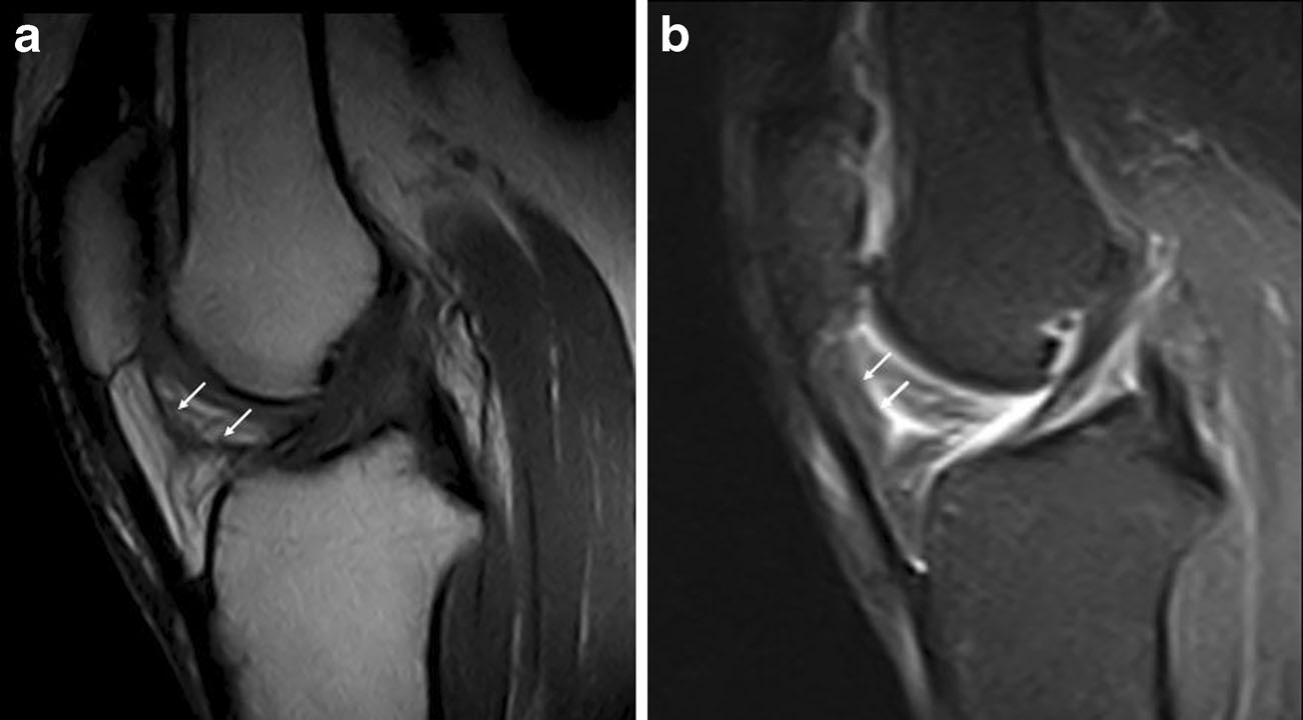
Infrapatellar thickened plica. Sagittal T1w (**a**) and proton density with fat saturation (**b**) MR images show thickened infrapatellar plica (*arrows*) with associated oedema of HFP

HFP is made of fat lobules separated by thin fibrous cords. It also contains a number of larger septae—such as the infrapatellar plica (Fig. 3)—which run from the intercondylar notch of the femur anteriorly through the fat pad and may reach the inferior pole of the patella [5–7]. Also known as the ligamentum mucosum, it is a normal anatomical structure and it represents remnants of synovial membranes from embryological development. It is the most common plica in the knee [5] and sometimes it can become symptomatic.

The normal vascular supply of the HFP consists of two vertical arteries located posterior to the lateral edges of the patellar tendon. They are branches of the superior and inferior genicular arteries (Fig. 1c) and are interconnected by two or three horizontal anastomotic arteries running inside the HFP. An additional anastomotic artery may be present within the infrapatellar synovial fold connecting the medial genicular artery and the middle or superior horizontal artery. While the peripheral area of HFP is well supplied, there is a paucity of vascularity in the central one [3].

The HFP is richly innervated—and is therefore a source of anterior knee pain. It receives branches of the femoral, common peroneal and saphenous nerves.

Although magnetic resonance imaging (MRI) of the knee can be realised with different sets of sequences and in some cases must be tailored on the clinical suspect, it generally included sagittal T1-weighted fast spin echo (FSE) (3.0-mm section thickness, field of view FOV 138 × 170 mm and matrix 208 × 512 pixels) and three-orthogonal-plane sequences with high contrast. High-contrast image such as short tau inversion recovery (STIR) or proton density (PD)-weighted with fat saturation can be employed depending on the employed machine. High-contrast images parameter can vary but some guidelines can be drawn (sagittal sequences, 3.0-mm section thickness, FOV 143 × 180 mm and matrix 204 × 512 pixels; coronal sequences, 3.0-mm section thickness, FOV 200 × 200 mm and matrix 204 × 512 pixels; axial 3.0-mm section thickness, FOV 180 × 162 mm and matrix 187 × 512 pixels).

A circular-polarised send-receive extremity coil is used. The acquisition time of any sequence ranges from around 3 min to 5 min 30 s.

At MRI, the HFP appears predominantly hyperintense on T1- and T2-weighted sequences (T1w and T2w sequences) and structurally similar to subcutaneous fat. The internal fibrous septa are hypointense on T1w sequence and hypointense in high-contrast sequences with fat saturation (T2w sequences with fat saturation, short inversion time inversion recovery [STIR]).

The infrapatellar plica is well imaged at MRI as a low-signal-intensity structure of variable size and thickness on T1w images [6] (Fig. 3a). It can be followed from its femoral origin—in the anterior part of the intercondylar notch—since its distal attachment into the inferior pole of the patella [5] (Fig. 3).

The HFP is a flexible, displaceable structure that accommodates to the different degrees of flexion-extension of the knee. The main function of HFP is to reduce friction between the patella, patellar tendon and deep skeletal structures. Moreover, it prevents pinching of the synovial membrane and it facilitates vascularisation of adjacent structures.

If painful impairment of knee function and oedema within the fat pad are associated with plica (Fig. 3b), plica syndrome may be diagnosed [2].

At the initial stage, non-surgical treatment involving physiotherapy and/or intraplical or intra-articular corticosteroid injections is preferable; however, symptomatic plicae may be treated with arthroscopic excision in recalcitrant cases [7].

### Pathogenesis of HFP disorders

#### Traumatic and post-traumatic disorders

**Fig. 4:**
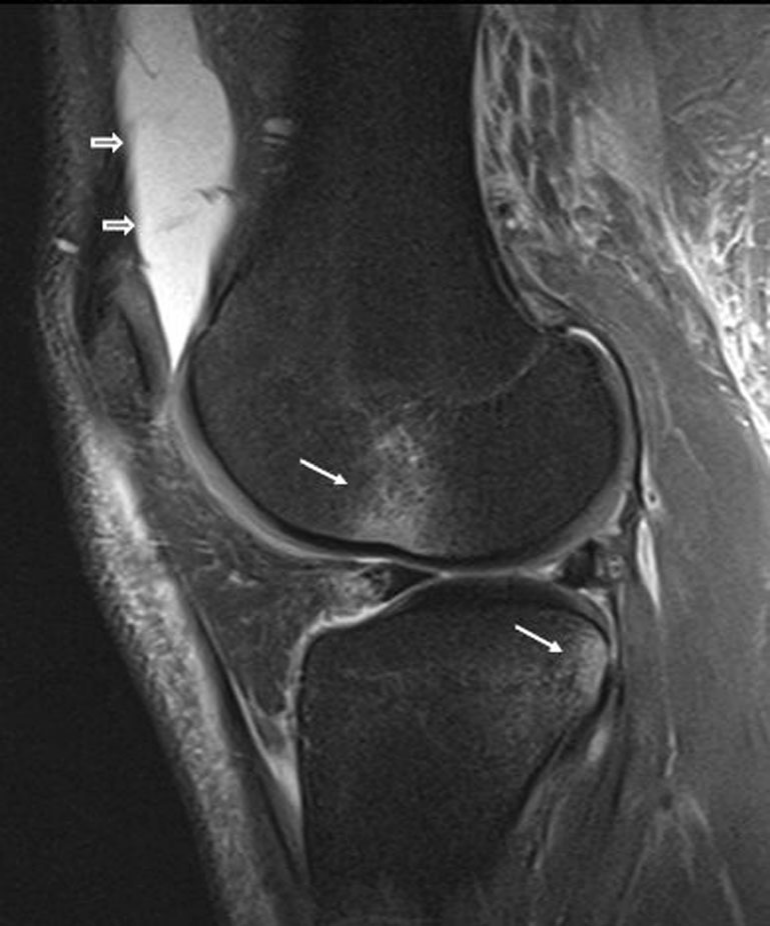
HFP acute trauma (pivot shift). MRI proton density with fat saturation image shows HFP apex oedema (*thin arrows*), oedema of the external femoral condyle and of the corresponding tibial plateau (*thin arrows*), with joint effusion (*wide arrows*)

**Fig. 5:**
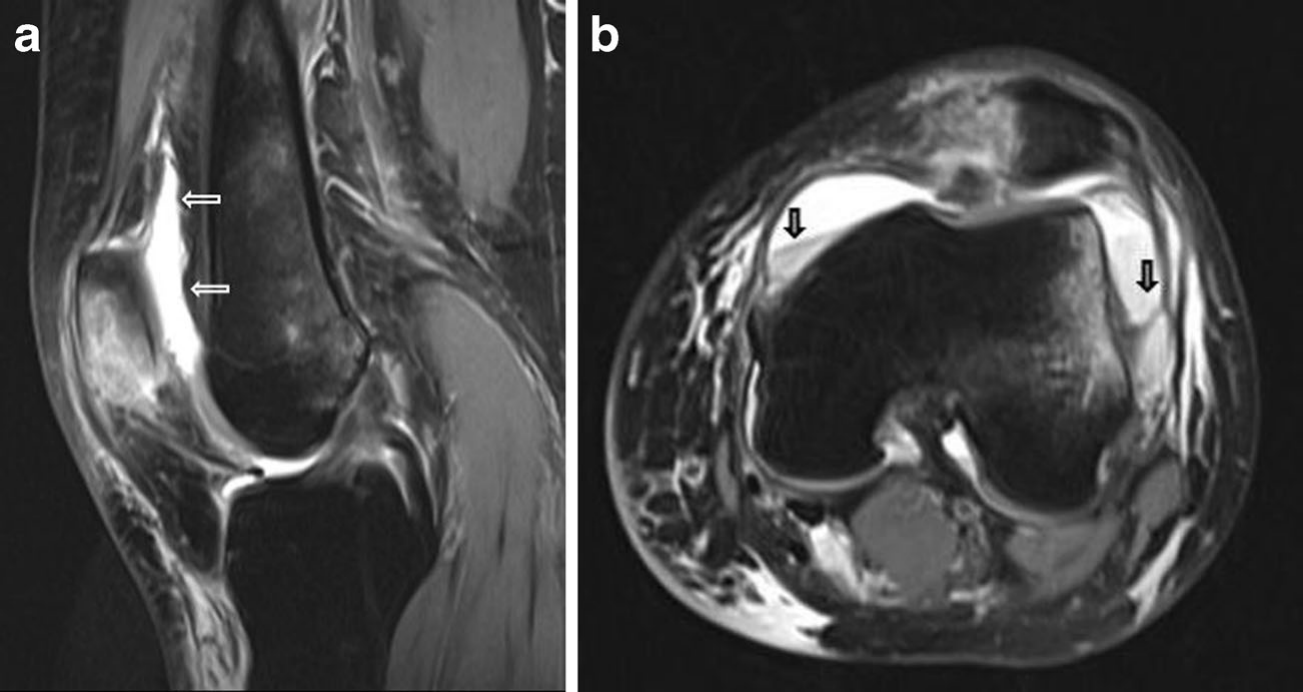
HFP acute trauma (direct contusion). MRI proton density with fat saturation images show HFP oedema, patellar oedema and joint effusion (*thin arrows*) (**a**) with fluid-fluid levels in axial image (*wide arrows*) related to hemarthrosis (**b**)

**Fig. 6:**
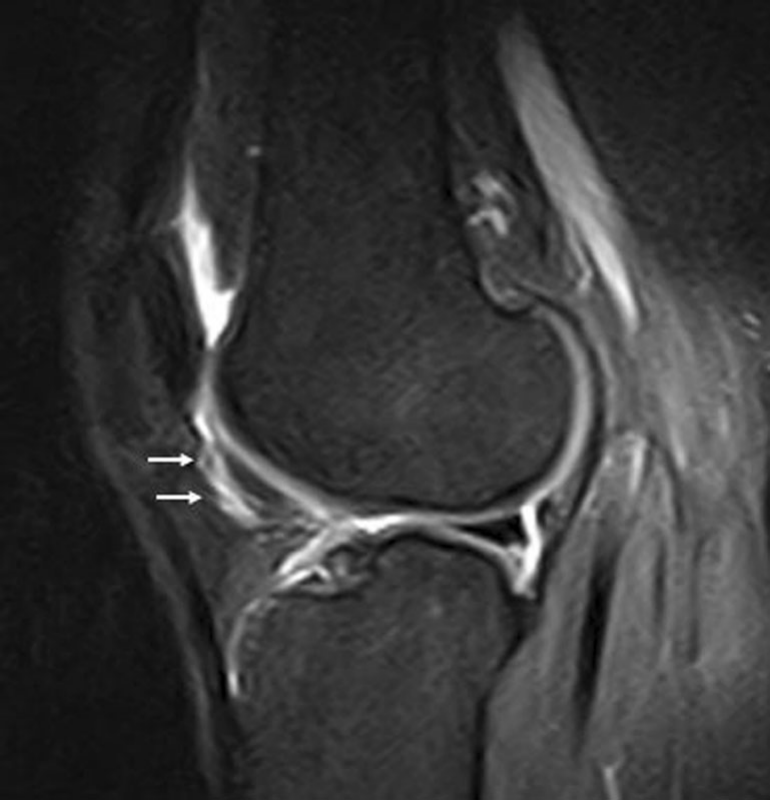
HFP acute trauma. MRI proton density with fat saturation image, after direct trauma, shows HFP fragmentation (*arrows*)

**Fig. 7:**
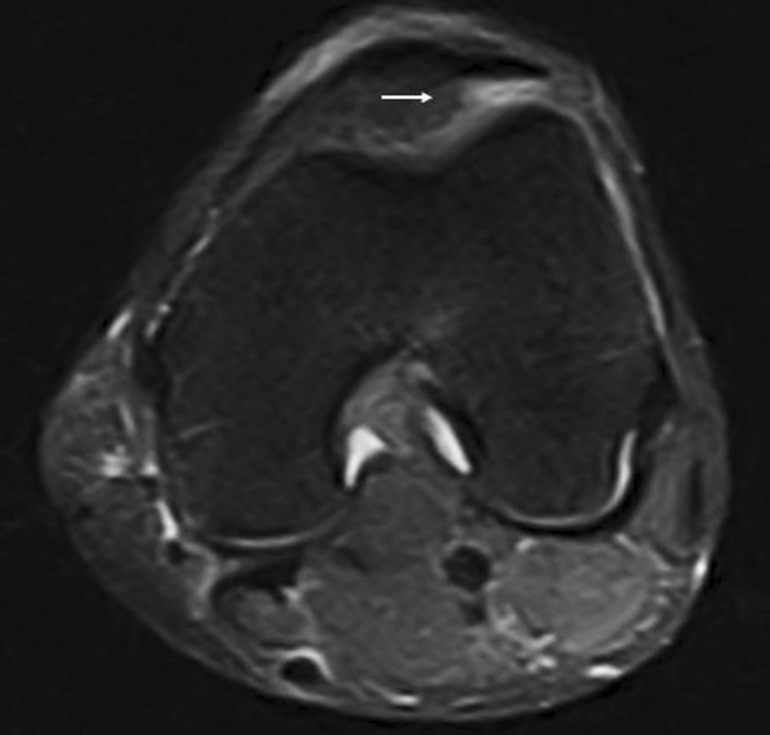
Superolateral HFP impingement. Axial proton density with fat saturation MR image shows oedema in superolateral portion of HFP in a young athlete with persistent knee pain in superolateral portion of the knee

**Fig. 8:**
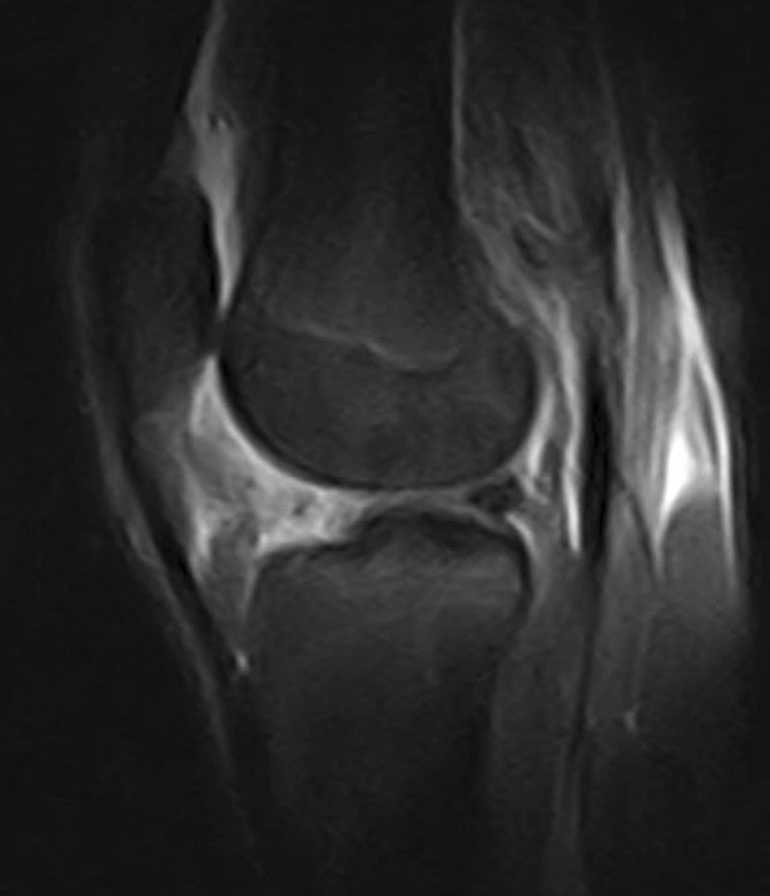
Hoffa’s disease. Repetitive microtraumas with hyperextension and rotational strain in soccer player. MRI proton density with fat saturation images demonstrates an enlarged, oedematous HFP

**Fig. 9:**
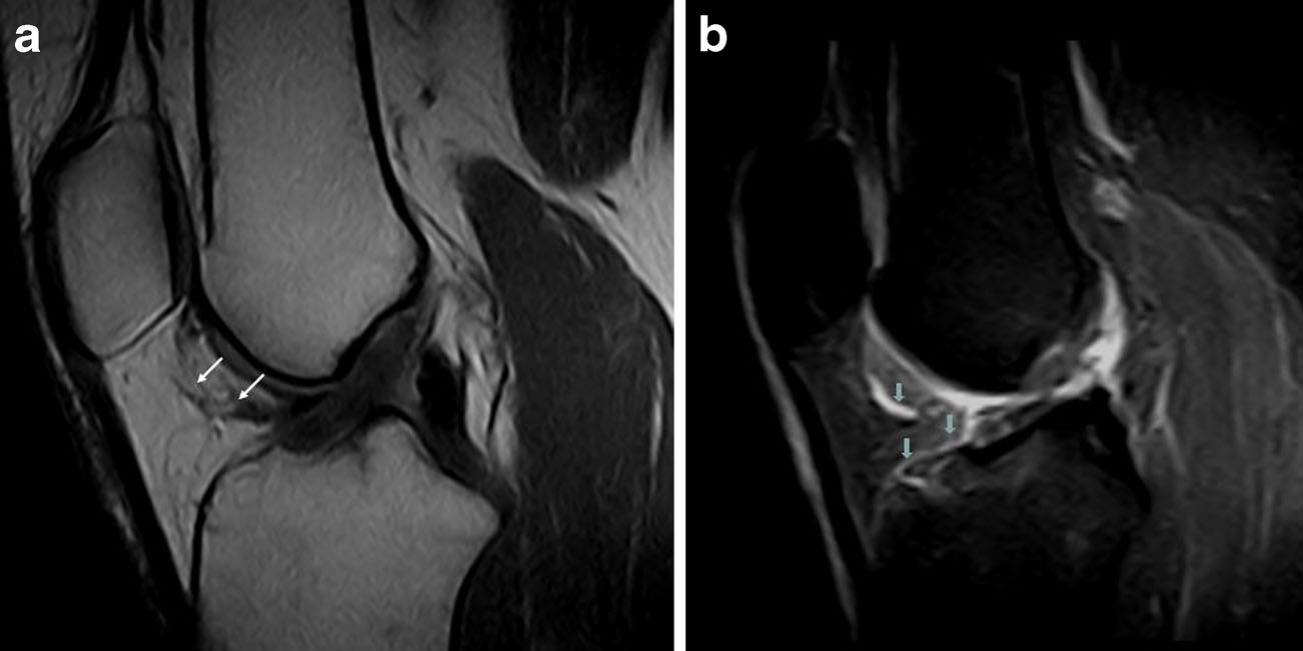
Post-arthroscopic fibrosis. Sagittal T1w image (**a**) post-arthroscopy shows scarring in the HFP (*arrows*) that is oedematous and hypervascularised (*arrows*) on proton density with fat saturation image (**b**)

**Fig. 10:**
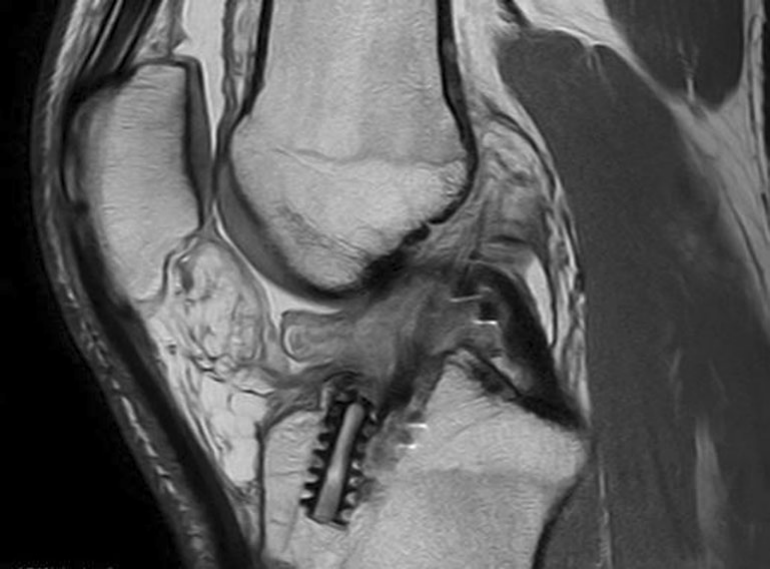
Cyclops lesion. Sagittal proton density image, post ACL reconstruction, shows hypointense mass in the fat pad, which is oedematous

**Fig. 11:**
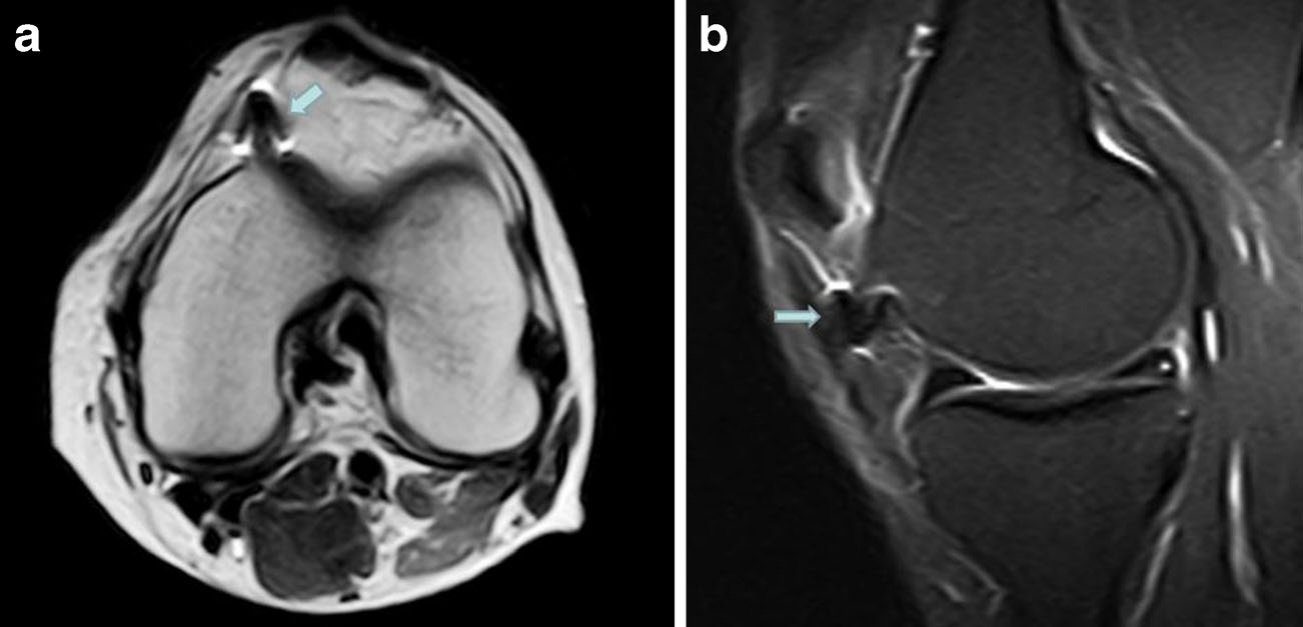
Post-surgical fibrosis. Axial T2w (**a**) and sagittal proton density with fat saturation (**b**) images show hypointense post-surgical fibrosis in the fat pad (*arrow*), which is oedematous

Since the main function of the HFP is to reduce friction between the patella, patellar tendon and deep skeletal structures it is not surprising that it can be injured in traumatic lesions involving the anterior knee. Disorders of HFP can follow indirect (Fig. 4) and direct (Figs. 5 and 6) acute traumas and chronic repetitive microtraumas, such as infrapatellar plica syndrome [5–7] or impingement syndrome [8–11] (Figs. 7 and 8). Acute traumas include also traumatic patellar dislocations [12–14] and surgical traumas [15–17]. Either acute or chronic, traumas can lead to local bleeding and inflammation—which are the most frequent causes of HFP-related pain—and eventually to a variety of arthrofibrotic lesions, such as post-traumatic or post-arthroscopic fibrosis (Fig. 9), “Cyclops” lesion (Fig. 10) and post-surgical fibrosis (Fig. 11) [17–20].

#### HFP lesions secondary to adjacent disorders

**Fig. 12:**
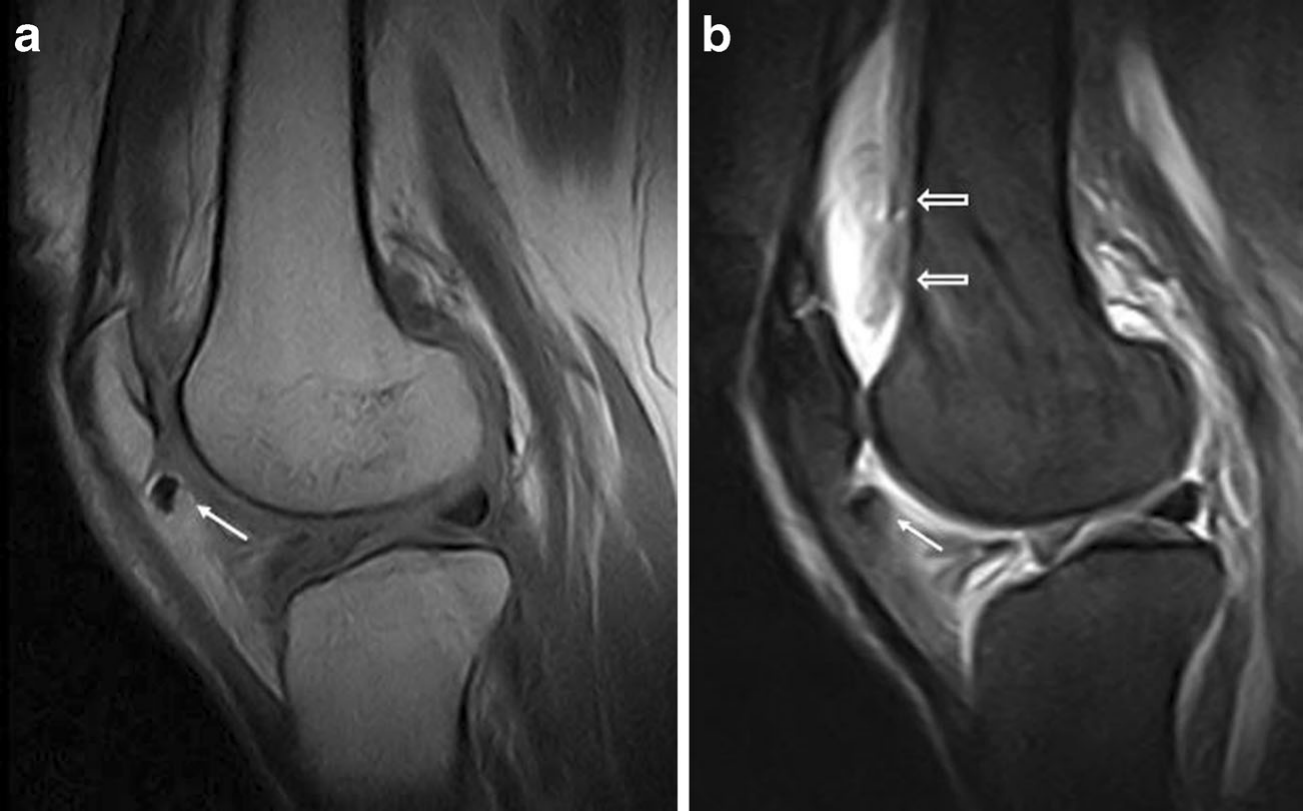
Lateral meniscal flap tear. Sagittal T1w image shows a fragment of the flap tear (*arrows*) displaced in the fat pad (**a**) that is oedematous on sagittal proton density with fat saturation image (**b**); joint effusion (*wide arrows*) is present

**Fig. 13:**
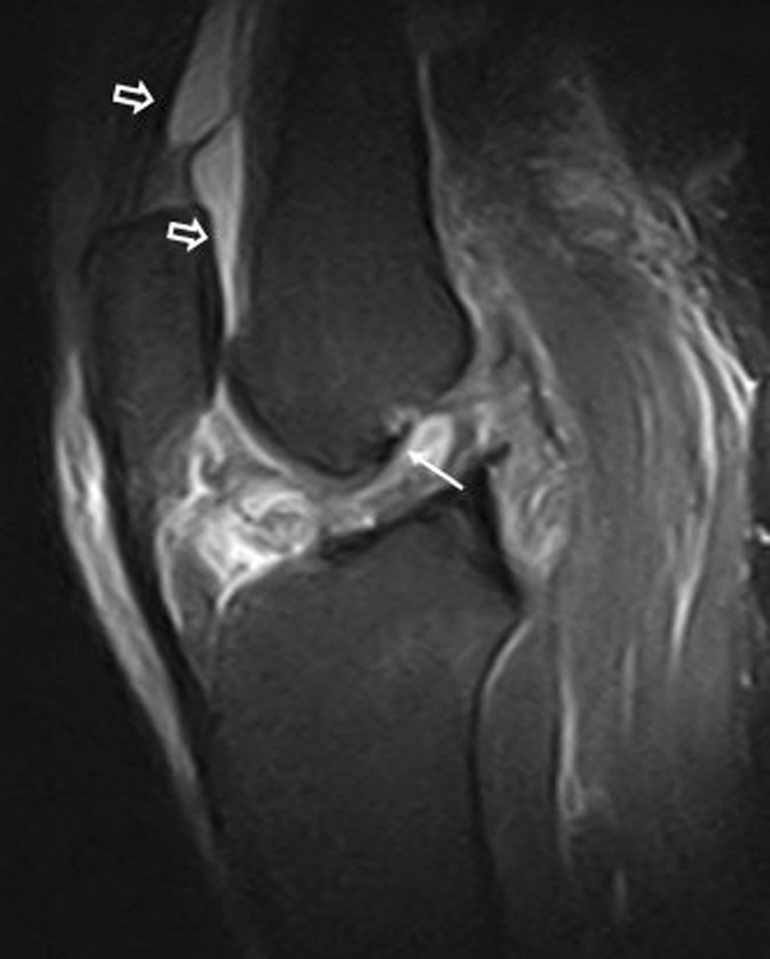
ACL tear. Sagittal proton density with fat saturation image shows ACL tear with residual femoral stump (*arrow*), joint effusion (*wide arrows*) and oedematous HFP

**Fig. 14:**
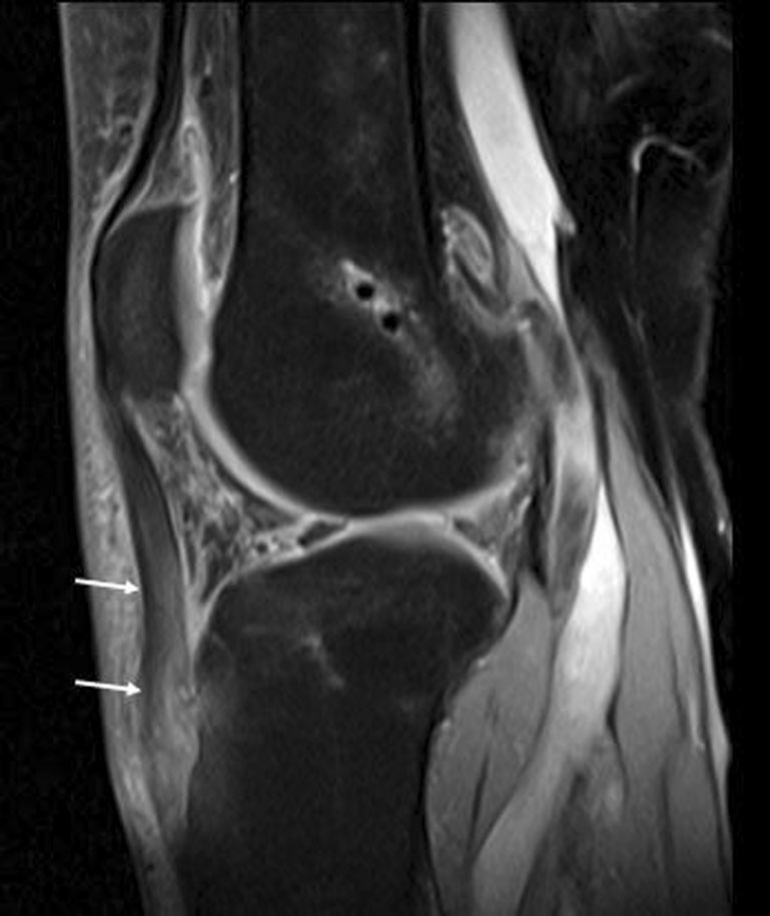
Patellar tendinopathy after autograft. Sagittal proton density with fat saturation image shows tendinopathy of patellar tendon (*arrows*) with increased signal intensity and increased anteroposterior diameter of the tendon, and oedema of the HFP

**Fig. 15:**
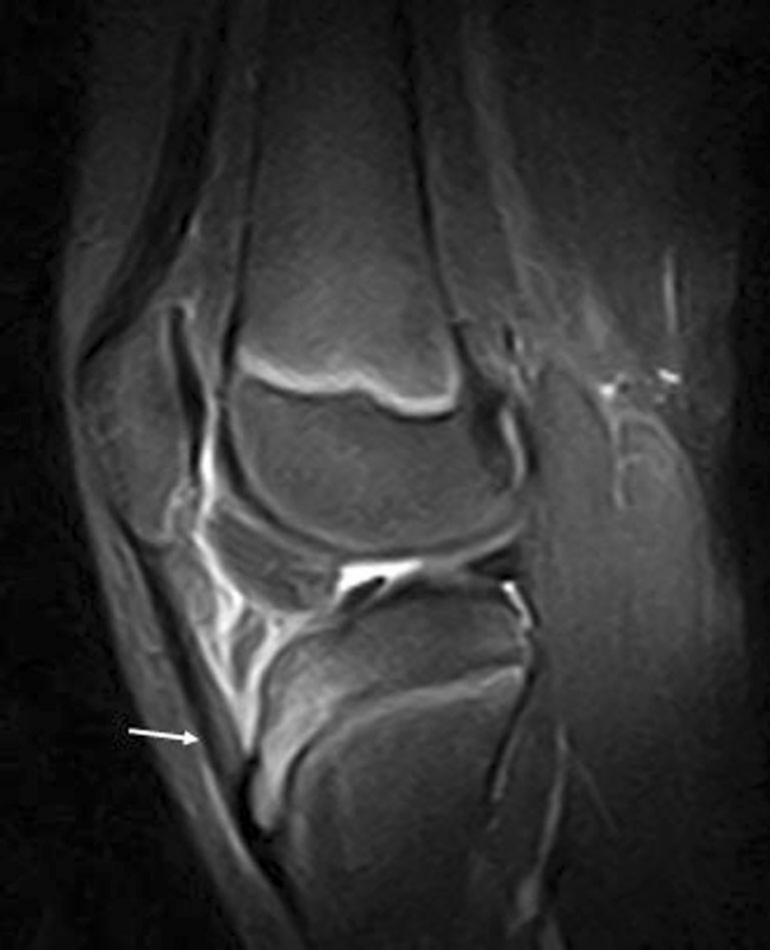
Osgood–Schlatter disease. Sagittal proton density with fat saturation image shows enlarged and oedematous patellar tendon insertion (*arrow*), bone marrow oedema of the tibial tuberosity and HFP oedema

**Fig. 16:**
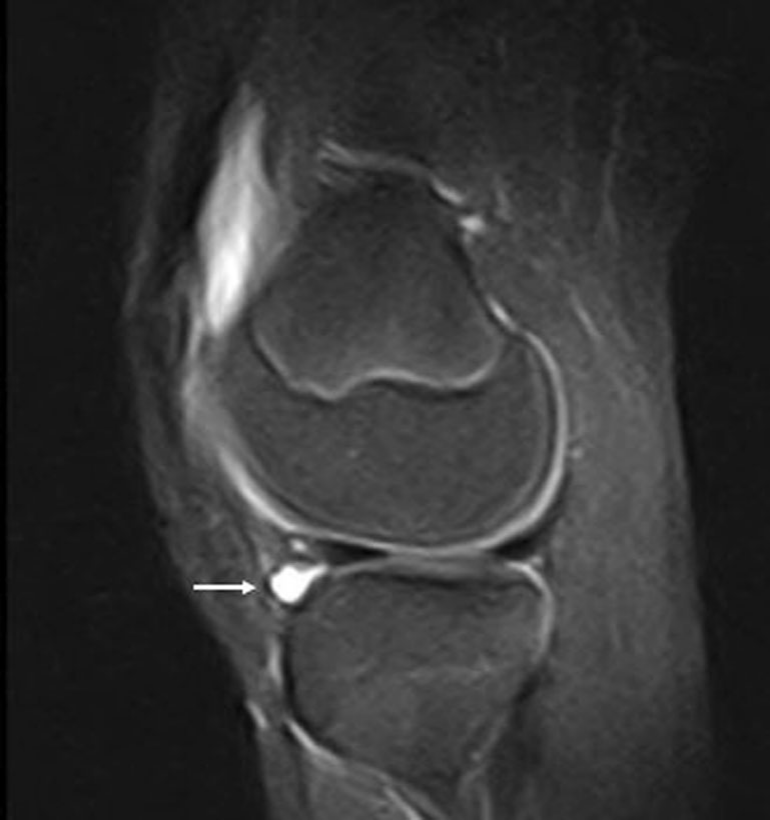
Meniscal cyst. Sagittal proton density with fat saturation image (**a**) shows meniscal cyst (*arrow*) extending into the fat pad (oedematous), arising from lateral meniscus

**Fig. 17:**
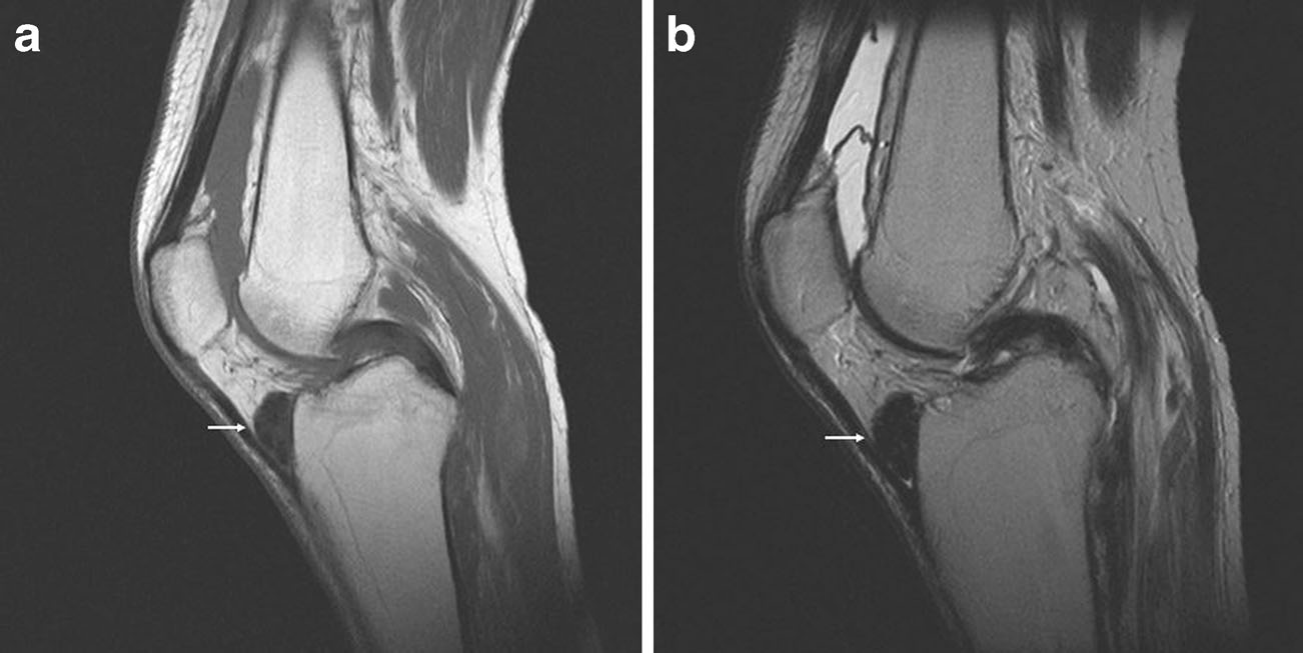
PVNS. Sagittal T1w (**a**) and T2w (**b**) images show synovial vegetation in the infrapatellar bursa (*arrows*); synovial vegetations are hypointense due to the paramagnetic effect of hemosiderin

**Fig. 18:**
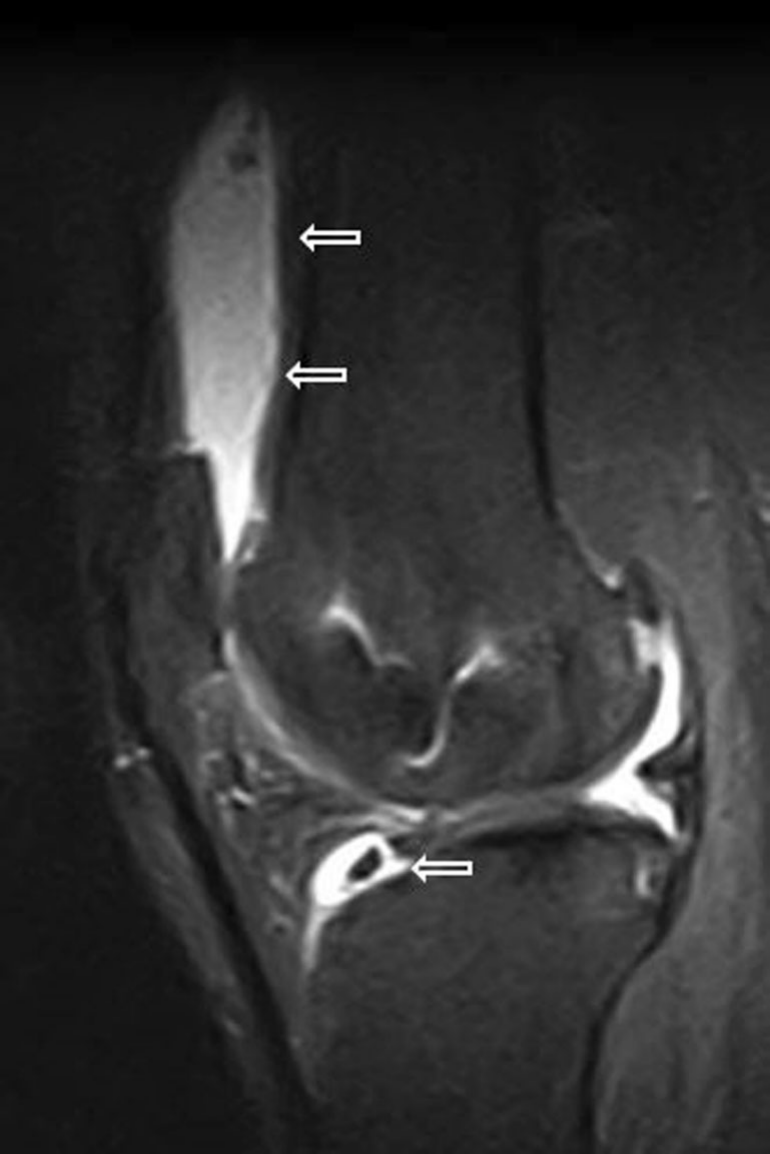
Synovial chondromatosis. Sagittal proton density with fat saturation image shows joint effusion with loose bodies in the suprapatellar pouch and in the infra-hoffatic recess (*wide arrows*). The HFP is oedematous

Due to its close relationship with several anatomical structures, the HFP can be secondarily involved by adjacent disorders. These include meniscal (Fig. 12) and ligamentous injuries [20] (Fig. 13), trauma of the patellar tendon [21–24] (Figs. 14 and 15), articular disorders, meniscal cysts [25–28] (Fig. 16) and synovial abnormalities [29–31] (Figs. 17 and 18).

#### Masses and pseudo-masses

**Fig. 19:**
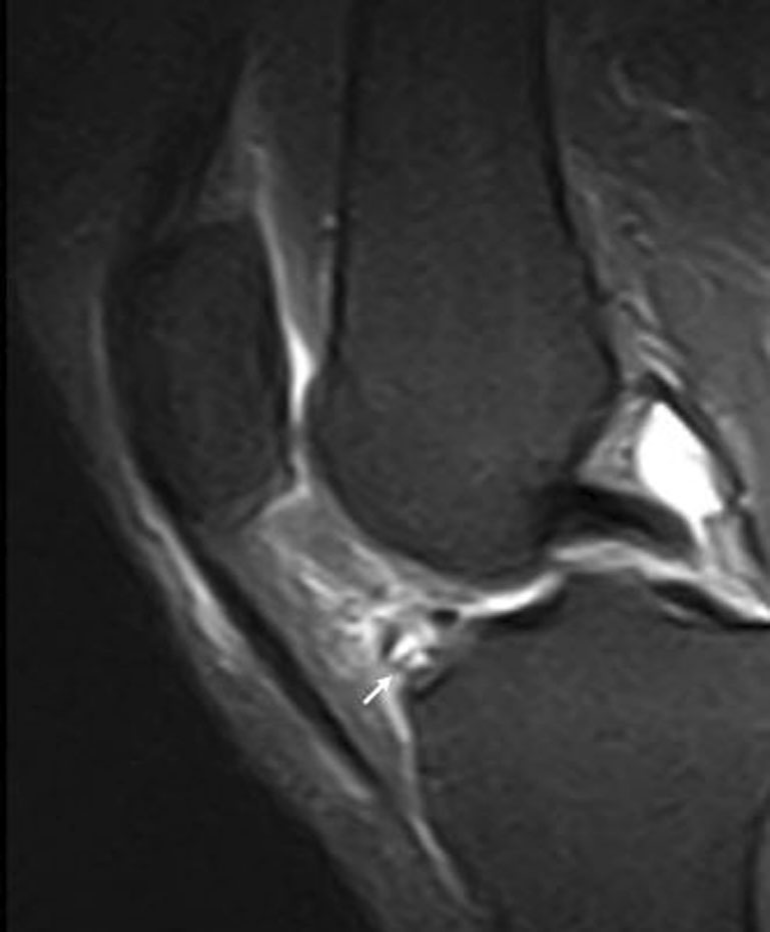
Ganglion. Sagittal proton density with fat saturation demonstrates a multilocular ganglion cyst (*arrows*) within HFP in a patient with anterior pain and oedema of the HFP

Masses or mass-like abnormalities rarely occur within the HFP, the most common being ganglia [32, 33] (Fig. 19).

Patients with oedema or abnormalities of the HFP on MRI are often symptomatic; however, oedema and abnormalities are also present in patients without symptoms. For this reason, a careful correlation with clinical findings is required to avoid unnecessary treatments in asymptomatic patients. The reason why some subjects do not feel pain, even though they present MRI findings which are similar to those in patients with local pain, is still unknown.

## Traumatic and post-traumatic disorders

An acute injury—via a variety of different mechanisms—may traumatise HFP, thus resulting in haemorrhagic (Fig. 5) or fibrous changes in HFP [29]. More often, traumas of the HFP are associated with injuries affecting other adjacent structures; this is likely in patellar fractures, sleeve fractures [14] and patellar dislocations. In these cases, the pathological findings of the HFP are less important than those affecting the adjacent structure and patients present symptoms related to the main trauma. Nevertheless, isolated lesions of the HFP are possible and are usually secondary to smaller trauma associated with compression of the fat pad between the femoral condyles and the tibial plateau [13] (Fig. 4). Post-traumatic lesions could include cleft formation and fragmentation of the fat pad (Fig. 6).

Acute oedema and haemorrhage characteristically manifest as areas of increased signal intensity on high-contrast MRI images (Figs. 4–6). Patients with traumatic oedema and/or fragmentation are symptomatic and present anterior knee pain.

In patients with associated acute trauma, treatment is mainly addressed to the adjacent pathology (e.g. patellar fractures, dislocations). On the other hand, in isolated lesions of the HFP a conservative treatment (local physiotherapy and non-steroidal anti-inflammatory drugs) is preferred.

### Impingement

HFP impingement is a clinical syndrome—manifesting mainly as anterior knee pain [8, 9]—which most commonly occurs after repetitive local microtraumas. Impingement is typically located at the superolateral portion of the HFP. It is secondary to repetitive pinch of the external portion of the fat pad between the lateral patellofemoral ligament and the cartilage of the lateral facet of the trochlea. Any conditions associated with a decrease in the distance between these two structures can facilitate a local impingement. A higher position of the patella (patella alta) is also associated with HFP impingement. The impingement is typically observed during full flexion of the knee. The patient presents lateral parapatellar pain aggravated by physical activity, which slowly disappears with rest. A joint effusion is only rarely detected. The differential diagnosis with chondromalacia of the patellar cartilage is difficult during the physical examination since the symptoms are nearly the same in both the conditions. Proximal patellar tendinopathy (jumper’s knee) presents anterior knee pain as well, and a local pressure of the proximal tendon reproduces the patient’s symptoms. Whereas ultrasound can easily confirm the diagnosis of jumper’s knee, this technique is useless in diagnosing HFP impingement.

Some studies have reported an association between HFP impingement and superolateral (Fig. 7), posterior or diffuse oedema (Fig. 8) [9, 11]. HFP oedema characteristically manifests as an area of increased signal intensity on high-contrast MRI images (Figs. 7 and 8). Intravenous contrast injection, which is rarely performed, shows local enhancement and the absence of local fluid collection. In chronic cases, the patellar cartilage can show thinning and localised erosions. Patients with microtraumatic oedema are symptomatic, but oedema may be an asymptomatic finding in patients without trauma or impingement [9]. As previously stated, a careful correlation between MRI data and the clinical findings is necessary to plan a correct treatment.

Treatments consist of physical therapy, taping, local injections of corticosteroid and avoiding sports associated with increase in local pain. Fat pad excision is reserved for patients with persistent pain after conservative treatment [9].

### Postoperative changes

HFP scars may result from previous surgery or arthroscopy [15–17] as an excessive fibrotic response during the repair process. Post-surgical fibrosis (Fig. 11) is usually ill-defined or confluent [15], while post-arthroscopic fibrosis (Fig. 9) generally manifests as bands coursing through the fat pad [15]. Post-surgical fibrotic changes within the HFP are usually asymptomatic. However, a careful investigation of other possible origins of the patient’s pain must be made before considering fibrosis of HFP as the cause of pain.

The Cyclops lesion (Fig. 10) is a nodular soft tissue mass that can complicate the reconstruction of the anterior cruciate ligament (ACL). The nodule is usually located in the posterior HFP, just anterior to the ligament graft [17], and it is named after the arthroscopic appearance of a Cyclops’ eye. Under a pathological point of view, nodules are made of fibrous tissues containing a central core of granulation tissue. Clinically, patients present local pain during activity and incomplete extension of the knee due to impingement of the nodule between the tibia and the inferior trochlea.

MRI is the imaging technique of choice to detect a Cyclops lesion. These lesions are best visualised in T1w sequences (Figs. 9a, 10 and 11a) in the sagittal plane, obtained at the level of the ACL graft. Examination at complete knee extension can confirm the local impingement of the nodule.

Knees subject to postoperative changes can have pain, generally associated with oedema (Figs. 9b and 11b) and a limited range of motion.

Treatment consists of physical therapy and injections of corticosteroid [18]. Patients with a lack of mobility show improvement in the range of motion thanks to the release of scar tissue and the removal of the fibrotic HFP performed either through surgery or—more often—through arthroscopy [19].

## HFP lesions secondary to adjacent disorders

Abnormalities of HFP—focal and diffuse oedema, tears and scars in particular—are more common in knees with meniscal (Fig. 12) or ACL tears (Fig. 13), or are associated with patellar tendon lesions.

Meniscal tears or acute injuries of the ACL may traumatise HFP; however, the most common alterations are focal oedema [20]—caused by the stretching or impingement of the fat pad between the femur and the tibia in patients with lesions of the ACL—and joint instability—characterised by posterior femoral translation in relation to the tibia. No additional treatment is necessary for changes of the HFP.

Patellar tendinopathies such as jumper’s knee [21], focal tendinopathy located on the lateral aspect of the patellar tendon [11, 22] and patellar tendon healing defect after patellar tendon autograft [23] might cause painful mechanical impingement of HFP [24] (Fig. 14).

Osgood-Schlatter disease typically occurs in young patients ranging from 10 to 14 years of age and it is caused by a repetitive traumatic traction of the patellar tendon performed on the immature tibial tubercle apophysis (which is still partially cartilaginous at that age). This traumatic mechanism causes cartilage swelling, fragmentation of the tibial tubercle ossification centre, patellar tendon lesions and reactive bursitis of the deep or superficial tibial infrapatellar bursae [25]. HFP may also be involved (Fig. 15).

Anterior knee pain is more common in patients with HFP oedema than in patients with only extrinsic lesions.

Treatment is addressed to the primary disease in patients with secondary involvement of the HFP due to adjacent disorders [12]. In meniscal or ACL tears, arthroscopy is suggested to symptomatic active patients after considering their lifestyle, the presence of knee catching or locking, and the sensation of “giving way” or joint instability in the sagittal plane. The presence of pathological changes inside the HFP does not change the treatment choice.

Meniscal cysts are fluid collections located within or adjacent to the meniscus (Fig. 16) and are caused by a fluid extravasation into the parameniscal soft tissue through a meniscal tear [25–28]. Meniscal tears have a horizontal component in most cases [28]. At MRI meniscal cysts are frequently septate; they present a fluid signal and are located close to a grade-3 meniscal lesion. Lobulated cysts adjacent to a normal meniscus are generally not meniscal cysts but ganglions. The HFP oedema that sometimes is adjacent to a meniscal cyst is probably secondary to leakage of the fluid through the cyst’s wall. At the initial stage, non-surgical treatment of meniscal cysts is preferable. Ultrasound–guided local steroid-lidocaine injections can lessen local inflammation and alleviate patient’s pain. In non-responding patients, surgical excision is indicated; in this case, meniscectomy may be necessary to prevent recurrence.

### Synovial pathology

Synovial pathologies, such as pigmented villonodular synovitis (PVNS) [3, 29, 30] (Fig. 17), synovial osteochondromatosis [3, 31] (Fig. 18), synovial hemangioma [3] and joint effusion [4], can be associated with focal or diffuse oedema of the HFP.

PVNS is a rare, articular diffuse or peri-articular focal, benign, synovial tissue proliferation. Detection of a diffuse hypertrophy of the synovial membrane at the anterior knee, or of a focal and well-circumscribed nodule, which shows hypointense signal in all MRI sequences (Fig. 17), is highly evocative of PVNS. The low signal intensity in the T2w images is due to the paramagnetic effect of hemosiderin contained inside the PVNS lesion [29].

Synovial osteochondromatosis is an idiopathic benign chondral and/or osseous metaplasia of the synovia. Its exact aetiology remains unknown and it presents loose bodies in the synovial cavity [31] (Fig. 18). Although standard radiographs can show radiopaque calcifications and intra-articular loose bodies, MRI is the imaging technique of choice in the accurate assessment of synovial osteochondromatosis.

The HFP oedema accompanying alterations of the synovial pathology is usually asymptomatic and does not need treatment. Treatment in cases of HFP involvement usually includes therapy for the various diseases.

## Masses or pseudo-masses

Ganglion cysts are benign masses containing a highly viscous fluid located within a dense fibrous connective tissue wall lacking a synovial lining. Ganglia can be unilocular or multilocular (Fig. 19), round or lobular, and communication between ganglia and adjacent joints is uncommon [3, 25–27]. Ganglia can produce pain and swelling, but they are usually asymptomatic [32]; when symptomatic they may require drainage or excision.

**Fig. 20:**
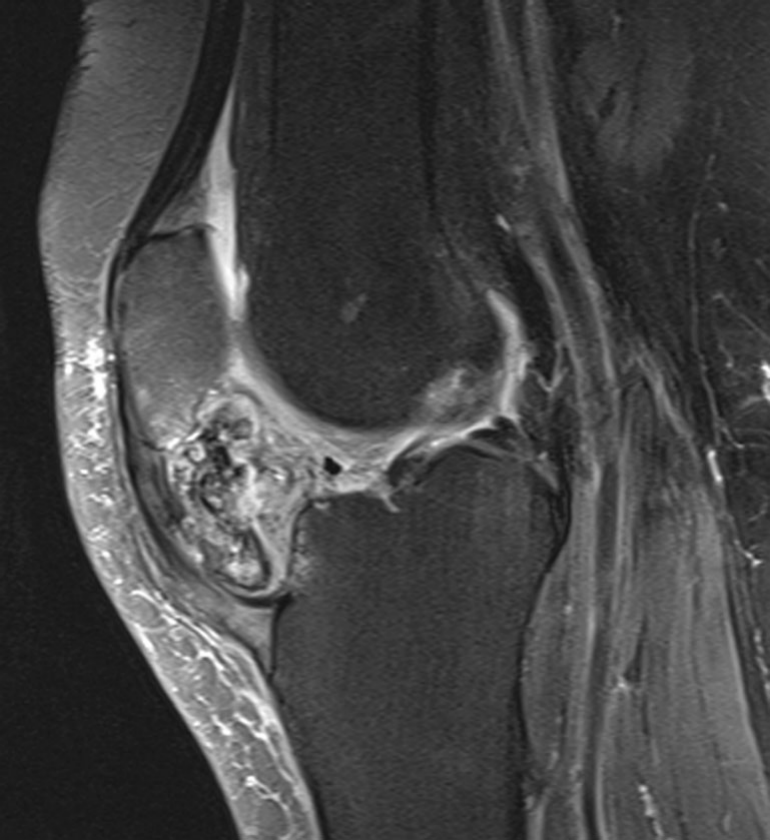
Extraskeletal chondroma, Sagittal proton density with fat saturation image shows a heterogeneous mass within the fat pad, with high signal of chondroid matrix and oedema and low signal of calcification or ossification (courtesy of Dr. L. Pietrobono, IRCCS Policlinico San Matteo, Pavia)

Chondroma is a result of metaplasia in capsule or adjacent connective tissues that leads to the deposition of osteoid material in HFP [34]. MRI high-contrast sequence images of the chondroma show a heterogeneous mass within the fat pad, with high signal of chondroid matrix and oedema, and low signal of calcification or ossification (Fig. 20). Persistent symptoms and treatment failure require arthroscopic or—more often—an open resection.

Synovial hemangioma is a benign vascular soft-tissue tumour, which frequently involves the knee. Clinical presentation is characterised by the presence of a circumscribed mass, which is covered by normal skin, increasing in size when the extremity is in the dependent position [3, 35]. MRI demonstrates a lobulated margin mass hypo- or iso-intense to muscle in T1w sequences and areas of confluent hyperintensity with discrete foci of hypo-intensity consistent with vessels and fibro-fatty septa in high-contrast sequence images. Open excision is the treatment of choice and results are good even for larger lesions.

## Conclusions

Acute injury and repetitive micro-trauma with a variety of different mechanisms could traumatise HFP, thus resulting in inflammatory, haemorrhagic and then fibrous changes. Post-traumatic lesions may include cleft formation and fragmentation.

MRI allows a detailed assessment of the normal anatomy of the HFP as well as of its main disorders. Acute oedema and haemorrhage characteristically manifest as areas of increased signal intensity on high-contrast MR images. Patients with post-traumatic or micro-traumatic oedema are usually symptomatic, but radiologists must be aware that HFP oedema may also be found in asymptomatic patients without trauma or impingement. A careful correlation with clinical data is necessary when interpreting MR images.

Patients with abnormalities of HFP—such as synovial plica without oedema—are generally pain-free, whereas those with lesions and oedema always suffer pain; patients with oedema not associated with other lesions frequently suffer pain, although there are cases of asymptomatic oedema. Radiologists should be cautious in emphasising abnormalities of HFP, since they do not always cause pain and/or difficulty in walking and, therefore, do not require therapy.
